# Cause Analysis of an Infection in Facelift Surgery Due to *Mycobacterium chelonae*

**DOI:** 10.3389/fmed.2019.00243

**Published:** 2019-11-07

**Authors:** Marie Decalonne, Emmanuel Lecorche, Estelle Hau, Agnès Petiteau, Célia Moreau, Odile Milan, Philipe Lanotte, Laurent Mereghetti, Emmanuelle Cambau, Nathalie van der Mee-Marquet

**Affiliations:** ^1^Centre d'Appui Pour la Prévention des Infections Associées aux Soins en Région Centre Val de Loire, Hôpital Bretonneau, Centre Hospitalier Universitaire, Tours, France; ^2^Centre National de Référence des Mycobactéries et de la Résistance des Mycobactéries aux Antituberculeux (CNR-MyRMA), APHP-Lariboisière Bactériologie, Université de Paris, INSERM UMR1137 IAME, Paris, France; ^3^Service de Dermatologie, Centre Hospitalier Universitaire Saint Louis, Paris, France; ^4^Service de Bactériologie-Virologie-Hygiène, Hôpital Bretonneau, Centre Hospitalier Universitaire, Tours, France

**Keywords:** *Mycobacterium chelonae*, WGS, post-surgical infection, tap water, showering

## Abstract

We report a post-facelift infection due to *Mycobacterium chelonae*. An environmental strain recovered from the water supply network of the surgical clinic and the clinical strains were considered non-differentiable using whole genome sequencing. After the unhealed wound's exposure to *M. chelonae* while showering early at the clinic after surgery, a lasting exposure of the colonized wound to the warm and moist working conditions of a bakery may have been favorable to the infection's development.

## Introduction

*Mycobacterium chelonae* is a ubiquitous, rapidly growing non-tuberculous mycobacterium that has been associated with post-operative infections (1–4). The investigations performed to inquire into the causes of these infections are rarely conclusive. Inadequate sterilization of surgical equipment and tap water have been suggested as sources of infection (5–9). We report a case of a post-facelift infection and present the findings of the root cause analysis performed in the course of this infection.

## Patient and Infection Description

A 63-year-old woman, heavy smoker with a significant medical history for prior breast cancer and a low body mass index (17.4 kg/m^2^), had a facelift in 2018 at a surgical clinic in the Centre region of France. She left the clinic at day 1 and was back to work at day 2 in a bakery. Signs of infection were not seen at day 10 when the stitches were removed. The disunion of the scar was observed at day 15, and small nodules appeared gradually during the next 3 months, first under the ears, then on the neck and cheeks. A microbiological analysis of the purulent secretions performed 3 months after the cosmetic procedure was performed. The samples were inoculated onto Coletsos medium (Bio-Rad, Marnes-la-Coquette France) and BBL MGIT + supplement (BD, Becton, Dickinson and Company, Sparks, USA), incubated for 3 months at 30 and 37°C. This microbiological analysis revealed *M. chelonae* infection. The patient was successfully treated with a polyantibiotherapy combining clarithromycin and tobramycine.

## Cause Analysis of the Post-surgical Infection

The post-surgical infection was subject to external national reporting, and a cause analysis was performed by the local and regional infection control teams and the surgeon.

The pre-, per-, and post-operative events were first reviewed. Two pre-operative showers were adequately performed at home by the patient the day before and the morning before surgery. By contrast, the pre-operative skin decolonization was not in line with the current French recommendations, as the antiseptic used was not an alcoholic but an aqueous solution of antiseptic. The per-operative antibiotic administration was adequate and performed more than 30 min before surgical incision (cefazolin 2 g). The operating time was not excessive (2 h 20 min). At day 1, the two surgical drains and the dressing were removed. There were no signs of infection. The same day, before leaving the clinic, and in contrast with the local and national recommendations, the patient had a shower with no protection of the wound. The wound was fully exposed to the water delivered by the water supply network from the surgical clinic. At day 2, the patient went back to work. The unhealed wound was then exposed to the moist and warm conditions of a bakery. In addition, the patient interview identified the use of diverse cosmetics on the scar since day 2.

Retrospective analysis of the microbiological data from the laboratory at the clinic did not give rise to any suspicion of additional cases of post-surgical infections during the past 12 months. The review of practices in the operating theater did not identify any hydric source that could have been responsible for a per-operative contamination of the wound. A research of mycobacteria in environmental samples was performed. All samples were inoculated onto Coletsos medium (Bio-Rad, Marnes-la-Coquette France) and BBL MGIT + supplement (BD, Becton, Dickinson and Company, Sparks, USA) and incubated for 4 weeks at 30 and 37°C.

Search for non-tuberculous mycobacteriae was negative for the patient's five cosmetic products (i.e., a de-make up lotion, micellar water, a day cream, sweet almond oil, and rose water). On the other hand, two water samples were collected: one from the shower in the patient's room and the second from the tap where the surgeons wash their hands before entering the operating rooms (1 L at each point). The two water samples were positive for different mycobacteria: a culture of *Mycobacterium kansasii* was recovered from the shower, and *M. chelonae, Mycobacterium paragordonae*, and *Mycobacterium llatzarense* were recovered from the tap water located in the operating theater.

## Genetic Characterization of the Clinical and Environmental Strains of *M. CHELONAE* (Figure 1)

*M. chelonae* isolates were sent to the French National Reference Center for Mycobacteria and Antimycobacterial Resistance. Identification was confirmed using GenoType NTM-DR 1.0 (Hain Lifescience). DNA were extracted by a DNA Ultraclean Microbial kit (QIAGEN, Hilden, Germany); DNA libraries were prepared with a Nextera XT kit (Illumina, San Diego, USA) and were sequenced with the MiSeq system (Illumina, San Diego, USA) and MiSeq Reagent V2 (2x150) kit (Illumina, San Diego, USA).

**Figure 1:**
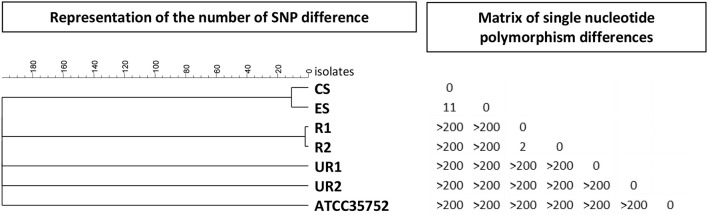
Comparison of SNPs from whole genome sequences of six *M. chelonae* isolates. Phylogenetic tree built with the unweighted pair group method with arithmetic mean (UPMGA) from 77,100 SNP positions found in the comparison of the seven isolates: clinical isolate obtained from the post-facelift infection (CS), environmental isolate from tap water at the surgical clinic (ES), epidemiologically related isolates of clinical and environmental origins (R1 and R2, respectively), epidemiologically unrelated isolates of clinical and environmental origins (UR1 and UR2, respectively) and ATCC35752.

Whole genome sequencing (WGS) was performed for six strains: two epidemiologically unrelated strains (UR1 and UR2) and two epidemiologically related strains (R1 and R2) previously studied by the national reference center for *Mycobacteriae*, the facelift infection strain (CS), and the strain recovered from tap water at the surgical clinic (ES). Whole genome analysis was generated by aligning sequencing reads of the isolates to the reference one reference strain (10) (ATCC 35752, NCBI Reference Sequence NZ_CP007220.1). Sequencing data were solved using Bionumerics version 7.6 (Applied Maths, Gent, Belgium). The reads were trimmed excluding base call with a Phred score below 15 and then aligned using the Trimming and Resequencing analysis options. The Single Nucleotide Polymorphism (SNP) signature was built using the Strict filtering (closed SNP set) option, retaining SNP with a minimum coverage of 5× at least covered once in both forward and reverse direction and a minimum distance between retained SNP position of 12 base pairs, and removing non-discriminatory position. The SNP matrix was used to build a maximum UMPGA tree.

The distance between the unrelated strains (UR1 and UR2) was >200 SNPs; the two related strains (R1 and R2) differed by two SNPs, and the clinical strain and the environmental strains studied for the present investigation differed by 11 SNPs (Figure 1). Based on the data currently available for *M. chelonae* species, the clinical and environmental strains were considered non-differentiable.

## Discussion

Nowadays, the source of surgical site contamination is usually endogenous, with microorganisms from the patient's cutaneous flora contaminating the incision site when the pre-operative skin decolonization is less than optimal. More rarely, the source of infection is exogenous, mostly with airborne or waterborne microorganisms contaminating the wound during the surgery of after surgery before healing. The genetic similarity of the clinical isolate and the isolate recovered from the water supply network of the clinic argues for a water source of contamination in this case of post-facelift infection. The contamination of the wound with *M. chelonae* may have occurred while showering at day 1. Unfortunately, we did not find *M. chelonae* into the water sample from the shower. However, (1) the recovery of *M. chelonae* from a tap water located in the operating theater likely indicated a global colonization of the water system from the clinic by *M. chelonae*; (2) the observation by the infection control team of several facelift surgical procedures and the careful study of the practices with the surgeon and its team did not reveal any change since at least a year, and did not recover any hydric source or aerosol used into the operating room; (3) the distance between the tap water where surgeons wash their hands and the operating room was too far away to allow direct contamination of the surgical site; and (4) the surgeon doesn't enter the operating room with wet hands, but performs hand antisepsis after washing his hands, and before entering the operating room where he gloves his hands.

There are many, varied risk factors of surgical site infection related to the patient (extreme age, diabetes, immunodepression, smoking, denutrition, obesity, and lack of education regarding post-surgical infection prevention issue), the surgery (urgency, surgeon's experience, foreign material, long-term surgery, and hemorrhagic surgery), the surgical and care teams (lack of education, low nurse/patient ratio, and high turnover), the pre-operative preparation of the patient (long-term hospitalization before surgery, lack of showering before surgery, and inadequate decolonization of the skin before incision), per-operative antibiotic administration, environmental contamination of the operating rooms, and the lack of asepsis during wound care before healing. In the present case, we believe that the infection occurred following contamination of the wound with *M. chelonae* while showering at day 1, favored by at least two contributing factors: the patient's low body mass index, and durable exposure of the non-healed, colonized wound to the moist, and warm conditions of the bakery from day 2.

## Conclusion

About one third of nosocomial infections are preventable (11). The strict application of the current recommendations for prevention of surgical site infection would have enabled us to prevent the occurrence of this case of post-facelift infection. If contamination of tap water with *M. chelonae* is inevitable, exposure of the wound to any non-sterile product (i.e., water, cosmetics) should be totally forbidden before wound healing. To improve the quality of care and to prevent additional cases of infection, a reminder of good practice was given to the surgeon and his team. Special attention should be paid to educating patients, so that they understand and respect this recommendation during the first days after surgery.

Sampling a large amount of water and WGS have been decisive for the root cause analysis of the infection. Our findings prove, if necessary, the usefulness of WGS for typing purposes in the *M. chelonae* species.

## Data Availability Statement

The datasets generated for this study are available on the National Center for Biotechnology Information website (https://www.ncbi.nlm.nih.gov/), BioProject number: PRJNA574109.

## Author Contributions

MD, AP, CM, OM, and NM-M performed the cause analysis. EL and EC supervised the isolation of *M. chelonae* from clinical and environmental samples and performed the molecular characterization of the strains using WGS. PL and LM supervised the isolation of *M. chelonae* from the water samples. EC and NM-M wrote the manuscript. EH performed the clinical diagnostic of the infection in first position.

### Conflict of Interest

The authors declare that the research was conducted in the absence of any commercial or financial relationships that could be construed as a potential conflict of interest.
